# Study protocol: the development of a randomised controlled trial testing a postcard intervention designed to reduce suicide risk among young help-seekers

**DOI:** 10.1186/1471-244X-9-59

**Published:** 2009-09-23

**Authors:** Jo Robinson, Sarah Hetrick, Sara Gook, Elizabeth Cosgrave, Hok Pan Yuen, Patrick McGorry, Alison Yung

**Affiliations:** 1 ORYGEN Youth Health Research Centre, Department of Psychiatry, University of Melbourne Locked Bag 10, 35 Poplar Road, Parkville, Victoria 3052, Australia

## Abstract

**Background:**

Suicidal behaviour and deliberate self harm are common among adolescents. Limited evidence exists regarding interventions that can reduce risk; however research indicates that maintaining contact either via letter or postcard with at-risk adults following discharge from services can reduce risk. The aim of the study is to test a postcard intervention among people aged 15-24 who presented to mental health services but are not accepted, yet are at risk of suicide.

**Methods/design:**

The study is a 3-year randomised controlled trial conducted at Orygen Youth Health Research Centre in Melbourne Australia. Participants are young help-seekers aged 15-24 who are at risk of suicide. Participants will be recruited over a 12 month period. The intervention comprises a regular postcard to be sent monthly for 12 months. The postcard enquires after their well being and includes information regarding individual sources of help and evidence-based self help strategies. Participants are assessed at baseline, 12 and 18 months.

**Discussion:**

This paper describes the development of a study which aims to reduce suicide risk in a sample of young help-seekers. If effective, this intervention could have significant clinical and research implications for a population who can be hard to treat and difficult to research.

**Trial Registration:**

The study was registered with the Australian Clinical Trials Registry; number: ACTRN012606000274572.

## Background

Suicidal and self-harming behaviour, including suicidal ideation, are common amongst adolescents. Among secondary school students, approximately 5-7% report having engaged in deliberate self-harm (DSH) in a 12 month period, whilst lifetime rates are 12-13% [1,2]. As many as 60% reported suicidal ideation [3].

Suicidal behaviour, is associated with a range of negative outcomes, most notably risk of further suicidal behaviour and completed suicide [4,5] and other forms of premature mortality such as accidental death and homicide [6]. A key risk factor for suicidal behaviour is mental disorder [7], particularly affective disorders and/or depressive symptoms [7,2]. However, mental disorders are not present in all young people at risk and suicidal behaviour is often precipitated by adverse life events or interpersonal crises [7].

Whilst much is known about the epidemiology of suicidal behaviour, less evidence exists regarding interventions to reduce risk [8], particularly that from randomised-controlled trials [9]. One promising intervention, tested in adults, has a focus on maintaining contact with those at risk. Two studies have shown that contact via regular letter or postcard with at-risk adults, after discharge from an inpatient psychiatric unit [10] or an Emergency Department (ED) [11] reduced subsequent suicidal behaviour for up to two years [10]. The treatment effect may be due to enhancing connectedness and perceived social support [12,11]. Treatment approaches that are successful among adults may not be among young people [13] and it is unknown whether the 'postcard' intervention would be successful with adolescents and young adults.

Research at a specialist mental health service (ORYGEN Research Centre, ORC) had showed that while a number of young people were not unwell enough to meet the entry criteria, this group included many individuals who had made suicide attempts (n = 14; 25%) or expressed suicidal ideation (n = 22; 38.6%) [14]. This highlighted a service provision gap that could potentially be filled by a postcard intervention.

## Methods/design

### Aims and hypotheses

The aims are to determine if suicidal and self harming behaviour can be reduced by the receipt of a regular postcard sent monthly over 1 year.

The hypotheses are that the receipt of a regular postcard will result in: 1. Decreased suicidal behaviour and ideation 2. A reduction in depression and hopelessness 3. Improved self-esteem 4. Increased perceived social support, and 5. Increased help seeking. We further hypothesise that: 6. There will be a positive association between reductions in suicidal behaviour and improved self-esteem and perceived support, and 7. Between reduced suicidal behaviour and hopelessness and depression.

### Study design

The study is a randomised controlled trial registered with the Australian Clinical Trials Registry and approved by the North Western Mental Health Research and Ethics Committee.

### Setting

Orygen Youth Health (OYH) is a publicly funded specialist mental health service for people aged 15-24 living in the Western and Northwestern regions of Melbourne. The service treats individuals with both psychotic and non-psychotic disorders. There is also a triage service, where eligibility for treatment is determined. It also houses Orygen Youth Health Research Centre (OYHRC).

### Participants

**Figure 1:**
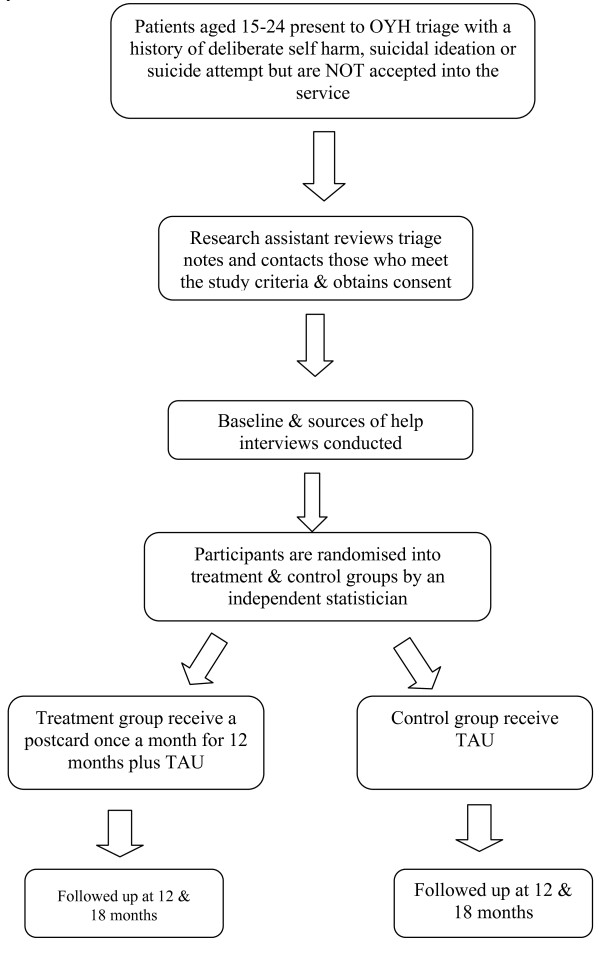
**Study flow-chart: This figure displays the participant flow-chart**.

Participants will be recruited from OYH triage over a 12 month period. Inclusion criteria are: 15 to 24 years; resident in Northwestern Metropolitan region of Melbourne; refused from service; current or lifetime history of suicidal ideation or attempts or DSH. These will be assessed by a Research Assistant (SG) via the written triage records. Exclusion criteria are: known organic cause for presentation; intellectual disability; inability to speak English. The participant flow chart is shown in Figure 1.

### Procedure

After assessing eligibility, the Research Assistant will telephone the young person to explain the study and seek informed consent. If the young person is interested in participating, the Research Assistant will arrange a face-to-face interview, during which written information will be provided and consent sought. Once consent is obtained the Research Assistant will conduct the baseline interview.

Following the baseline interview the study coordinator (JR) will contact each participant by telephone (within 1 to 5 days), enquire about wellbeing and ask him or her to identify 3 sources of help that may be useful in times of crisis. It will be explained that this information would be included in a postcard should the young person be allocated to the intervention group.

### Intervention

**Figure 2:**
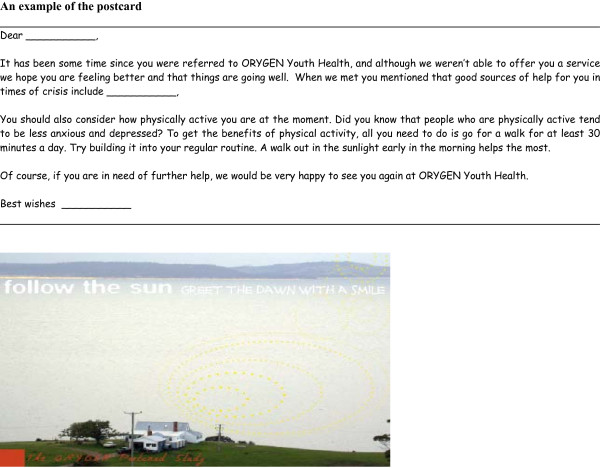
**Postcard: This figure contains an example of the postcard used in the study**.

The intervention is a regular postcard sent in a sealed envelope. One postcard a month is sent over 12 months, starting the month after the baseline assessment. The postcards have been designed with a 'youth focus', in conjunction with the OYHRC consumer group. They all enquire about the person's well being, remind them about the sources of help identified during the telephone interview and promote one of 6 evidence-based self-help strategies. These are: 1. Physical activity 2. Early morning light exposure 3. Self-help books based on cognitive behavioural therapy 4. Websites known to be effective e.g. BluePages [15] and Mood GYM [16] 5. Relaxation training 6. Reducing alcohol and other substance use [15-18]. The self-help strategies and the individual sources of help will be rotated. The participant's name and the individual source of help obtained from telephone interview will be hand-written and each postcard individually signed. An example of the postcard is shown in Figure 2.

Included with each postcard will be a 'change of address' slip, as well as the option for participants to request that the postcards be discontinued.

### Control intervention

Those in the control group will receive the initial 'sources of help' interview but no postcards.

### Outcomes

The primary outcomes are suicidal behaviour and ideation, DSH, hopelessness, depression, self-esteem, help-seeking and perceived social support, with assessment at baseline, 12 and 18-months. The acceptability of the intervention will also be assessed by calculating the proportion of participants who choose to discontinue receiving the postcards and via an evaluation questionnaire administered at 12-month follow-up. This questionnaire also asks participants whether or not they had employed any of the help-seeking strategies recommended in the postcards.

### Outcome measures

Basic demographic details, including age, gender, employment or educational status, living circumstances, country of birth, parents' or guardians' employment status, parents' or guardians' country of birth, medical history and details of any treatment being received, are recorded on a specifically designed, standardised questionnaire.

Axis-I diagnoses will be assessed using the Structured Clinical Interview for DSM-IV [19].

Depression will be assessed by the Center for Epidemiologic Studies - Depression scale [20] a 20-item measure of current level of depressive symptomatology. A cut off score of 24 was used in this study in order to detect symptoms more likely to be of clinical significance [21].

The Beck Scale for Suicidal Ideation (BSS) [22] measures suicidal ideation and intent. This is a 19 item self-report scale with items rated on a 3-point scale (0-2). The total score ranges from 0 to 38. Higher scores indicate greater suicidal ideation.

The Brief Reason for Living Inventory - Adolescent Version (BRFL-A) [23] is a self-report instrument that assesses suicide ideation and behaviour. Higher scores, as rated on a 6-point scale (1 = not at all important to 6 = extremely important), indicate greater reasons for living.

The Suicidal Behaviours Questionnaire (SBQ-14) [24] measures five domains: past suicidal ideation, future suicidal ideation, past suicide threats, future suicide attempts, and the likelihood of dying in a future suicide attempt. These domains are rated across a number of time points: (1) the past several days including today, (2) past month, (3) past four months, (4) past year, and (5) lifetime. The SBQ-14 also assesses the number of episodes of DSH and suicide intent during the past year. In the present study, this section of the questionnaire was modified to assess lifetime and past month ratings of self-injurious behaviour and suicide intent in order to provide more specific information about chronicity and current risk of suicidal behaviour.

The Beck Hopelessness Scale [25] measures hopelessness. This is a 20-item true-false scale that measures the extent of negative attitudes about the future. A total score is obtained by summing the ratings with a potential scoring range of 0-20. High scores reflect greater hopelessness.

The Rosenberg Self-Esteem scale [26] is a 10-item measure of global self-worth. Statements are rated on Likert scale ranging from strongly agree (1) to strongly disagree (4), with a total scale range of 10-40. Low versus high self esteem was dichotomized using a cut-off score of 20, with scores of below 20 indicating high self-esteem and scores of 20 or above indicating low self-esteem.

The Multidimensional Scale of Perceived Social Support [27] measures perceived social support. This 12-item instrument includes items rated on a 7-point scale ranging from "very strongly agree" to "very strongly disagree". The total score ranges from 12-84 with a higher score indicating a greater level of perceived social support.

The General and Actual Help Seeking Scales [28] measure help-seeking intentions, appraising both formal and informal sources. Participants rate help-seeking intentions ranging from 1 ("extremely unlikely") to 7 ("extremely likely") for each help source option including "no one". Higher scores indicate higher intentions. The AHSQ measures actual help-seeking behaviour. In this study participants were asked whether help had been sought for "an emotional problem" within the last two weeks. Generally, this measure is reported as three subscales: whether or not informal help has been sought; whether or not formal help has been sought; and whether or not any help has been sought.

### Sample size calculation: effect size and statistical power

Suicide and SA rates from other studies vary between 3.9% and 15.1% for a contact group, and 4.6% and 17.3% for a non-contact group [11,10], with small effect sizes. The design of our study differs from previous studies and it is not possible to make any firm inference for our study, although it is reasonable to expect a similar effect size. The power analysis for this study is based on using the general linear model to compare the intervention and control groups with the baseline values of an outcome measure as the covariate. It is assumed (conservatively) that the covariate would explain 5% of the variance in the dependent variable. Based on previous research at this centre, we expect to recruit 180 subjects who would be equally allocated to the two groups and conservatively estimated a drop-out rate of 20%. A power calculation indicated that we would be able to detect a small effect size of 0.21 with a significance level of 0.05 and power of 0.8. The actual sample size (n = 165) has made only a small change to the power calculation - the detectable effect size has increased from 0.21 to 0.22.

### Randomisation/treatment allocation

Random allocation to the postcard group and the control group will be carried out by the independent statistician (HPY), who has no knowledge about participants characteristics, using blocked randomization and computer generated random numbers. This will be concealed from the research team. The statistician will notify the study coordinator regarding the group allocation. The study coordinator will send out the postcards once per month.

The research assistant, who carries out the assessments, will be blind to group allocation. The success of blinding will be assessed at 18-months via questioning the research assistant regarding whether they think the participant received postcards as part of the RCT - Yes/No.

### Statistical methods

At each follow-up time point the general linear model will be used to test for the treatment factor, i.e. to compare the intervention and control groups for each outcome measure. The corresponding baseline values of each outcome measure will be used as the covariate. In addition, the effects of other possible predictors (such as gender and age) on outcome will be explored. Multi-level modeling will be used to compare the trend over time for each outcome in the two groups. Both the last observation carried forward and multiple imputation techniques will be considered if the amount of missing values is substantial.

### Safety and supervision

The research assistant received training in the administration of the measures and in the assessment of suicide risk. The research assistant will make contact with the research fellow (JR) overseeing the study once each assessment was complete. If there is no cause for concern, contact will be made via SMS. If there are concerns about the participant, contact will be made by telephone or face-to-face and the concerns will be discussed. If the research fellow is concerned about the participant, an immediate referral will be made to an appropriate service. Additionally, weekly supervision meetings with an experienced clinical psychologist (EC) will be held, during which all cases will be presented and any diagnostic and/or risk issues discussed. Any young people considered to be at elevated risk will be referred to an appropriate service.

## Discussion

This paper describes the protocol for a RCT that aims to reduce suicide risk among young help-seekers. Although much is known about the epidemiology of suicide in young people, we know less about effective interventions, and there are few studies that have successfully tested interventions via an RCT [10]. One of the reasons for this lack of evidence is that people at risk of suicide are often excluded from trials because of the ethical implications of denying treatment to this population [9]. However, if the postcard intervention is shown to be effective it could be used as a control treatment when testing other, more intensive interventions, hence allowing an at-risk population to participate in future trials. A further difficulty highlighted by previous research is that studies testing interventions designed to reduce suicide risk are often hampered by small sample sizes [11,29,10]. Again, because of the nature of the postcard intervention, it could be tested in large samples and in a range of settings.

If effective, this low-cost and transferable intervention has the potential to reach large numbers of people who are traditionally hard to engage in treatment. It is well documented that young people at risk of suicide are often non-compliant with medication and frequently disengage from services [30]. Therefore this intervention, whilst clearly no substitute for treatment, may be a useful supplement, or alternative when people refuse other forms of treatment or do not meet the criteria for specialist care. It could also be used following discharge from services, a time when risk is known to be elevated [31].

## Competing interests

The authors declare that they have no competing interests.

## Authors' contributions

Authors JR, AY, PM and HPY were all involved in conception of the study and made substantial contributions to the study design. In addition SG, EC made significant contributions to acquisition of data, training and clinical supervision. All authors have been involved in drafting the manuscript and have seen and approved the final version.

## Pre-publication history

The pre-publication history for this paper can be accessed here:


